# Data from a proteomic analysis highlight different osmoadaptations in two strain of *Propionibacterium freudenreichii*

**DOI:** 10.1016/j.dib.2019.104932

**Published:** 2019-12-04

**Authors:** Floriane Gaucher, Sylvie Bonnassie, Houem Rabah, Pauline Leverrier, Sandrine Pottier, Julien Jardin, Valérie Briard-Bion, Pierre Marchand, Romain Jeantet, Philippe Blanc, Gwénaël Jan

**Affiliations:** a UMR STLO, Agrocampus Ouest, INRA, F-35042, Rennes, France; b Bioprox, 6 rue Barbès, 92532, Levallois-Perret, France; c Université de Rennes I, Univ. Rennes, Rennes, France; d Bba, Pôle Agronomique Ouest, Régions Bretagne et Pays de la Loire, F-35042 Rennes, France; e de Duve Institute, Université catholique de Louvain, Avenue Hippocrate 75, Brussels, 1200, Belgium; f Univ. Rennes, CNRS, ISCR - UMR 6226, PRISM, BIOSIT - UMS 3480, F-35000, Rennes, France

**Keywords:** Propionibacterium, Osmotic, Stress, Probiotic, Osmoadaptation, Proteomic, Cheese, Osmoprotectant

## Abstract

The article presents a proteomic data set generated by a comparative analysis of the proteomes of *Propionibacterium freudenreichii,* comparing the CIRM-BIA 129 and CIRM-BIA 1025 strains. The two strains were cultivated until the beginning of the stationary phase in a chemical defined medium (MMO), and in this medium in the presence of NaCl, with or without glycine betaine. Whole-cell proteins were extracted, trypsinolyzed and analyzed by nano LC-MS/MS, prior to identification and classification by function using the X!Tandem pipeline software and the proteomic data from NCBI.nlm.nigh.gov. Quantification of proteins was then carried out in order to detect change in their expression depending on the culture medium. This article is related to the research article entitled “Benefits and drawbacks of osmotic adjustment in *Propionibacterium freudenreichii*”. The comparative proteomic analysis of the two strains reveal strain-dependent and medium-dependent stress proteomes in the probiotic *P. freudenreichii*.

**Table:** Specifications Table

Subject area	Microbiology, Food technology
More specific subject area	Probiotics, dairy starters
Type of data	Table and figure
How data was acquired	Mass spectrometry
Data format	Raw, Analyzed and Filtrated
Experimental factors	*Propionibacterium freudenreichii* CIRM-BIA 129 and CIRM-BIA 1025 were grown in chemically defined medium, and in presence of salt with or without glycine betaine. Whole cell proteins were extracted and analyzed by nanoLC-MS/MS in a gel-free strategy.
Experimental features	All the samples were analyzed by nano-LC coupled to MS/MS
Data source location	Both strains of *P. freudenreichii*, isolated from French cheeses, are the property of CNIEL, France. Data were acquired at INRA STLO, France, under the agreement of an MTA.
Data accessibility	Data have been deposit into an INRA data base: https://doi.org/10.15454/VVNQHR
Related research article	Gaucher F., Bonnassie S, Rabah H, Leverrier P, Pottier, S, Jardin J, Briard-Brion V, Marchand P, Jeantet R, Blanc, P, Jan G, Benefits and drawbacks of osmotic adjustment in *Propionibacterium freudenreichii,* Journal of proteomics (2019). In press

**Table:** 

**Value of the Data** • This dataset is an important step in understanding the molecular mechanisms responsible for osmoadaptation in P. freudenreichii. • A chemically defined medium was developed to clearly distinguish the effect of 1) osmotic constraint imposed by salt and 2) osmoadaptation in the presence of the universal osmoprotectants glycine betaine in a probiotic bacterium. • Surprisingly, molecular mechanisms of osmoadaptation are shown here to vary among strains within the same species.

## 1 Data

**Fig. 1:**
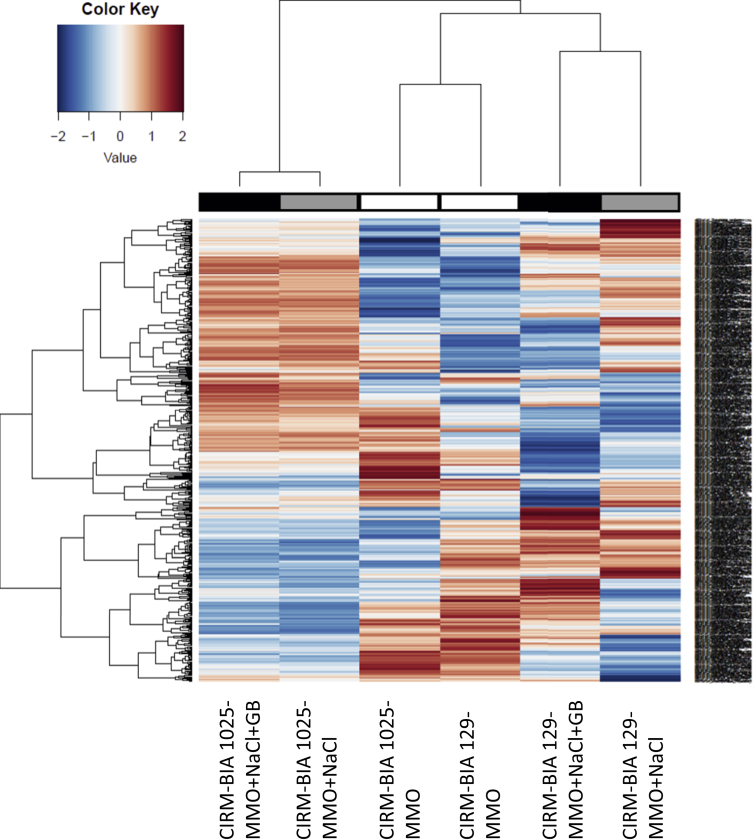
Heatmap representing variations in relative abundance of all detected proteins, ranging from -2 (blue) to +2 (red). Analysed proteomes were extracted from *P. freudenreichii* CIRM-BIA 129 or CIRM-BIA 1025, cultivated in different growth medium: MMO, MMO+NaCl, MMO+NaCl+GB. Proteins were extracted, identified and quantified as described in materials and methods.

This article presents a dataset generated during a proteomic comparative analysis of the cellular proteome of two phenotypical different *Propionibacterium freudenreichii* strains (CIRM-BIA 129 and CIRM-BIA 1025). The list of all proteins identified is provided in Supplementary Table 1, which presents the protein relative abundances. Fig. 1 shows a heatmap corresponding to abundances, for the two strains and the three culture media (chemically defined medium, the same with salt, the same with salt and glycine betaine). This report presents data from the research article entitled “Benefits and drawbacks of osmotic adjustment in *Propionibacterium freudenreichii*”. The cellular proteomes reveal diversity in the osmoadaptation of *P. freudenreichii*, among strains.

The proteomic data acquired during this study are presented in Supplementary Table 1 which summarizes all the mean values and all protein with a ratio above 1.5 or below 0.66. For each biological and each technical replicate, a raw data file is presented (https://doi.org/10.15454/VVNQHR).

Fig. 1 summarizes all the observed inductions and repressions. It is composed of a heatmap corresponding to abundances, for the two strains and the three culture media (chemically defined medium, the same with salt, the same with salt and glycine betaine).

## 2 Experimental design, materials, and methods

### 2.1 Strain and pre-culture

*Propionibacterium freudenreichii* CIRM-BIA 129 (equivalent ITG P20) and CIRM-BIA 1025 (equivalent ITG P1) were provided, stored and maintained by the CIRM-BIA Biological Resource Center (Centre International de Ressources Microbiennes-Bactéries d’Intérêt Alimentaire, INRA, Rennes, France). The strains are routinely cultivated in yeast extract lactate (YEL) medium [1] in this study, they are also cultivated in MMO (Medium Minus Osmoprotectants). MMO is a synthetic medium which was derivated from CdM (Chemiclal defined Medium) describe previously [2], MMO medium has the same composition as CdM but glutamate, glutamine and proline, which are considered as potent sources of osmoprotectants, were removed. *P. freudenreichii* was grown at 30 °C without agitation under microphilic condition. Composition of MMO is detailed in Supplementary Table 1.

### 2.2 Whole cell protein extraction and trypsin digestion

The label free proteomics has have been conducted as Huang et al., 2018 [3]. At the beginning of stationary phase, *P. freudenreichii* cells were harvested by centrifugation and washed twice with PBS buffer (NaCl 8g.L^−1^, KCl 2g.L^−1^ KH_2_PO_4_ 2g.L^−1^, Na_2_HPO_4_ 12H_2_O 35,8g.L^−1^). Cell pellets were then re-suspended in lysis solution (lysis solution: 0.5 mL pH 7.5, 157g Tris-HCl, 0.03 g SDS, 0.3 g DTT and 9.5 mL H_2_O), with 1 mM phenylmethylsulfonyl fluoride (PMSF, Sigma-Aldrich, USA). The solution was frozen for 1 hour, then sonicated (2 min 30 HZ), and cells were broken using zirconium beads in the homogenizer (Homogénéisateur Precellys Evolution - Bertin Instruments, France). The resulting SDS extracts were recovered by centrifugation (21,000 ×*g*; 20 °C; 20 min) and then cleaned and quantified using the two-dimensional (2-D) clean-up kit (GE Healthcare Bio-Sciences AB, Uppsala, Sweden) and the 2-D quant kit (GE Healthcare Bio-Sciences AB, Uppsala, Sweden), respectively. Tryptic digestion was performed on 100 μg of whole-cell proteins from each sample during 15 hours at 37 °C using Sequencing Grade Modified Trypsin (Promega, Madison, USA) according to the manufacturer's instructions and as described previously [3]. Spectrophotometric-grade trifluoroacetic acid (TFA) (Sigma-Aldrich, USA) was added in order to stop the digestion.

### 2.3 Nano LC-MS/MS

Experiments were performed as previously described [3]. Experiments were performed using a nano RSLC Dionex U3000 system fitted to a Q-Exactive mass spectrometer (Thermo Scientific, San Jose, USA) equipped with a nano-electrospray ion source. A preliminary sample concentration step was performed on a C18 pepMap100 reverse phase column (C18 column, 300-μm inner diameter (i.d.), 5 mm length, 5 μm particle size, 100 Å pore size; Dionex, Amsterdam, The Netherlands). Peptides separation was performed on a reversed-phase column (PepMap 100 C18, 75 μm i.d., 250 mm length, 3 μm particle size, 100 Å pore size; Dionex, Amsterdam, The Netherlands) with a column temperature of 35 °C, using solvent A (2% (v/v) acetonitrile (Honeywell, USA), 0.08% (v/v) formic acid and 0.01% (v/v) TFA in deionized water) and solvent B (95% (v/v) acetonitrile, 0.08% (v/v) formic acid and 0.01% (v/v) TFA in deionized water). Peptides were separated using a gradient of 5–35% solvent B over 80 min followed by 35–85% solvent B over 5 min at a flow rate of 0.3 μL/min. Eluted peptides were directly electro sprayed into the mass spectrometer operating in positive ion mode with a voltage of 2kV. Spectra were recorded in full MS mode and selected in a mass range 250–2000 *m*/*z* for MS spectra with a resolution of 70,000 at *m*/*z* 200. For each scan, the ten most intense ions were selected for fragmentation. MS/MS spectra were recorded with a resolution of 17,500 at *m*/*z* 200 and the parent ion was subsequently excluded from MS/MS fragmentation for 20 s. The instrument was externally calibrated according to the supplier's instructions.

### 2.4 Proteins identification

Proteins identification was performed as previously described [3]. Peptides were identified from the MS/MS spectra using the X!Tandem pipeline software (Langella et al., 2017). The search was performed against a database composed of proteomes of strains *P. freudenreichii* CIRM-BIA 129 and CIRM-BIA 1025 (downloaded from NCBI.nlm.nih.gov on the August 23, 2018). Database search parameters were specified as follow: trypsin cleavage was used and the peptide mass tolerance was set to 10 ppm for MS and 0.05 Da for MS/MS. Oxidation of methionine and phosphorylation of threonine, serine and tryptophan were selected as a variable modification. For each peptide identified, a minimum score corresponding to an e-value below 0.05 was considered as a prerequisite for peptide validation.

### 2.5 Protein quantification

Protein quantification was performed as previously described [3]. Every peptide identified by tandem mass spectrometry was quantified using the free MassChroQ software (Valot et al., 2011) before data treatment and statistical analysis within the R software (R 3.2.2, Project for statistical computing). A specific R package called ‘MassChroqR’ was used to automatically filter dubious peptides for which standard deviation of retention time was superior to 30 s and to regroup peptide quantification data into proteins. Two different and complementary methods of analysis were used, based on peak counting or XIC (eXtracted Ion Current). For peak counting analysis, variance analysis was performed to detect significant abundance variation between culture conditions. Proteins with an adjusted p-value <0.05 were considered significantly different. For XIC based quantification, normalization was performed to take into account possible global quantitative variations between LC-MS runs. Peptides shared between different proteins were automatically excluded from the data set as well as peptides present in less than 95% of samples. Missing data were then imputed from a linear regression based on other peptide intensities for the same protein [4]. Protein abundance was computed as the sum of peptide areas requiring a minimum of 2 peptides per protein. For every missing protein abundance, missing value were replaced by the minimum abundance obtained for this protein in the whole experiment. Analysis of variance was used to determine proteins with significantly different abundance between our culture conditions [5].
